# Modulation of TRIB3 and Macrophage Phenotype to Attenuate Insulin Resistance After Downhill Running in Mice

**DOI:** 10.3389/fphys.2021.637432

**Published:** 2021-06-09

**Authors:** Wei Luo, Yue Zhou, Qiang Tang, Lei Ai, Yuan Zhang

**Affiliations:** ^1^Department of Sports and Health Sciences, Nanjing Sport Institute, Nanjing, China; ^2^Department of Exercise Physiology, Beijing Sport University, Beijing, China; ^3^Jiangsu Research Institute of Sports Science, Nanjing, China

**Keywords:** insulin resistance, eccentric exercise, diet regulation, macrophage, TRIB3-AKT signaling

## Abstract

Eccentric exercise training accompanied by a low-fat diet can prevent insulin resistance (IR) and is currently an effective method for the treatment of IR induced by high-fat diet (HFD)-associated obesity. However, the molecular mechanisms underlying this improvement of IR in adipose tissue are still not completely clear. In this study, 5–6-week-old male mice were randomly divided into a standard control diet (SCD) group (SC, *n* = 12) and a HFD group (HF, *n* = 72). After 12 weeks, 12 mice in each group were randomly sacrificed. The remaining mice in the HF group were randomly submitted to one of the following experimental protocols for 8 weeks: obesity-HFD-sedentary (OHF-Sed, *n* = 14), obesity-HFD-exercise (OHF-Ex, *n* = 16), obesity-SCD-sedentary (OSC-Sed, *n* = 14), and obesity-SCD-exercise (OSC-Ex, *n* = 16). All obese mice in the exercise group were subjected to downhill running. Half of the mice in each group received an insulin injection (0.75 U/kg) before sample collection. Epididymal fat was removed and weighed. Adipocyte size and inflammatory cell infiltration were observed by H&E staining. Both basal and insulin-stimulated GLUT4 fluorescence and protein contents were detected by immunofluorescence and Western blot. Levels of IL-1β and IL-10 were detected by ELISA. Protein contents of iNOS, Arg-1, TRIB3, p-AKT, and AKT were determined by Western blot. CD86 and CD206 fluorescence were determined by immunofluorescence. The results showed that a HFD for 12 weeks induced IR accompanied by adipose tissue macrophages M1 polarization (increased iNOS protein content and CD86 fluorescence) and TRIB3-AKT activation. Downhill running accompanied by a low-fat diet attenuated IR (*p* < 0.01), reduced inflammation levels (increased IL-10 protein content and decreased IL-1β protein content), inhibited adipose tissue macrophages M1 polarization (decreased iNOS protein content and CD86 fluorescence) and promoted M2 polarization (increased Arg-1 protein content and CD206 fluorescence), and suppressed TRIB3-AKT signaling. We concluded that downhill running accompanied by dietary fat regulation attenuates HFD-related IR in mice, which may be associated with reduced TRIB3-AKT signaling and activated M2 macrophages in adipose tissue.

## Introduction

Excessive caloric intake and lack of physical activity are major contributors to the development of obesity-related metabolism diseases, insulin resistance (IR) is widely viewed as a common basis of the etiology of these metabolism diseases (Katula et al., 2013). Adipose tissue as the largest energy reservoir and endocrine organ is essential for the maintenance of systemic glucose, lipid, and energy homeostasis. Adipose tissue dysfunction is a hallmark of metabolic disorders and has been observed in obesity, IR, and diabetes (Liu et al., 2020). Insulin sensitivity in adipose tissue is critical to the maintenance of euglycemia by inhibiting triglyceride lipolysis and activating glucose uptakes in adipocytes. In the context of obesity, insulin sensitivity in adipose tissue is impaired and is associated with macrophage infiltration and inflammation (Kubota et al., 2018; Petersen and Shulman, 2018). Although the etiology underlying obesity-related IR is complex, it is well accepted that M1 macrophage accumulation in adipose tissue and the resulting local low-grade inflammation are mainly causation events in the pathogenesis of IR occurring with obesity (Castoldi et al., 2016; Gómez-Hernández et al., 2016).

Macrophages infiltrating into adipose tissue lie along a continuum and orientate toward either predominantly pro-inflammatory (M1) or anti-inflammatory (M2) phenotypes (Lumeng et al., 2007). M1 macrophages accumulate with obesity and secrete pro-inflammatory cytokines such as interleukin-1β (IL-1β) and tumor necrosis factor-α (TNF-α) (Amano et al., 2014; Pincu et al., 2015; Batra et al., 2018), and are characterized by the presence of inducible nitric oxide synthase (iNOS) and cluster of differentiation 86 (CD86) (Chen et al., 2018; Liu et al., 2018), which have been directly linked to IR (Appari et al., 2018). M2 macrophages are predominant in the adipose tissue of healthy lean individuals and produce anti-inflammatory cytokines such as IL-10 (Shapouri-Moghaddam et al., 2018), and are characterized by the presence of arginase-1 (Arg-1) (Chen et al., 2018) and cluster of differentiation 206 (CD206) (Zhang et al., 2017).

Exercise training accompanied by a low-fat diet can prevent IR and is currently an effective method for the treatment of IR induced by high-fat diet (HFD)-associated obesity (Luo et al., 2020). Compared with concentric exercises, eccentric exercise performed at the same mechanical power induces larger increases in post-exercise resting energy expenditure, partially because of the increase in muscle protein turnover and gain in lean mass (Julian et al., 2018, 2019). Eccentric exercise can also modify metabolic substrate use by increasing post-exercise fat oxidation and reducing glucose oxidation, leading to a switch to a more oxidative metabolism (Julian et al., 2018, 2019). In line with the increased demand of the working muscle for fatty acid substrates to regenerate injured muscles, eccentric exercise improves the blood lipid profile to a greater extent than concentric exercises (Paschalis et al., 2011; Julian et al., 2018, 2019). The improvement in insulin sensitivity after chronic eccentric exercise may also result from increased fat oxidation, which decrease chronic low-grade inflammation (Paschalis et al., 2011; Chen et al., 2017; Julian et al., 2019). Although the mechanisms of eccentric exercise which promote muscle hypertrophy to improve IR have been popularly studied, the metabolic mechanisms that could improve insulin sensitivity in adipose tissue need more investigation (Julian et al., 2019). Recently, downhill running has been conceived and characterized as “continuous moderate-load eccentric exercise,” which is characterized by lower metabolic and cardiorespiratory demands than concentric exercise when performed at the same power output. Thus, downhill running is a promising exercise modality for chronic metabolic diseases in patients who often experience dyspnea during classical concentric exercises (Julian et al., 2018). In addition, it has been reported that the conjunction of exercise and dietary regulation is more efficient in treating metabolic syndrome when compared with exercise training or dietary regulation alone (Oh et al., 2014; Normandin et al., 2017). As a result, the current investigation used downhill running accompanied by a standard control diet (SCD, 12% of energy from fat) to explore the potential mechanism of exercise training and dietary regulation that may attenuate adipose tissue IR and inflammation.

Tribbles homology protein 3 (TRIB3), also named as TRB3, SIKP3, and NIPK, is a pseudokinase that suppresses AKT activation by occupying its phosphorylation site (Zhang et al., 2016). As an important stress-related gene, TRIB3 is related to various signals such as endoplasmic reticulum stress and nutrient availability in adipose tissue, skeletal muscle, and the liver (Prudente et al., 2012). The over-expression of TRIB3 in the liver results in an increase of hepatic glucose output and higher blood glucose levels (Du et al., 2003). TRIB3 expression is elevated in skeletal muscle of IR patients and hyperglycemic mice, and are positively correlated with fasting blood glucose (Liu et al., 2010). These results suggest that TRIB3-AKT signaling has a critical role in regulating glucose homeostasis and could mediate IR.

In the current study, HFD-induced obesity mice were used to investigate the effects of exercise training accompanied by dietary regulation on insulin sensitivity, macrophage phenotype, inflammation, and TRIB3-AKT signaling in adipose tissue. The data to verify HFD-induced IR in mice have been previously published (Luo et al., 2020), which demonstrated that a HFD for 12 weeks induced obesity accompanied with dyslipidemia and impaired glucoregulatory capacity. In this study, we hypothesized that downhill running accompanied by dietary fat regulation would suppress TRIB3-AKT signaling and promote macrophage M2 polarization, thus improving IR in adipose tissue.

## Materials and Methods

### Experimental Animals

**FIGURE 1:**
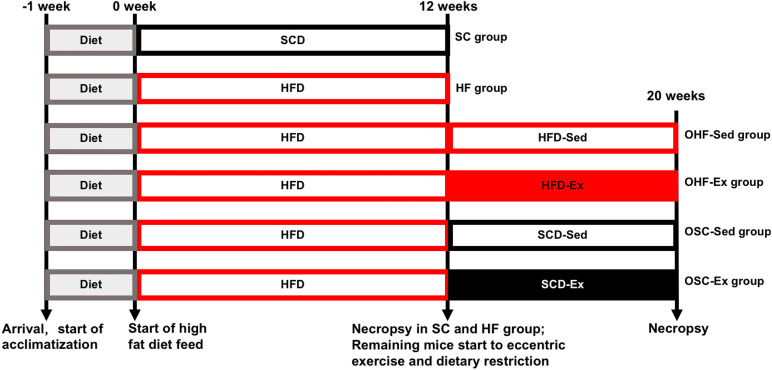
Overall design of this study. After 1 week of acclimatization, mice were fed a standard control diet (SC group) or high-fat diet (HF group) for 12 weeks. After 12 weeks of diet, 12 mice in each group were randomly sacrificed to detect the effect of the HFD. The remaining mice in the HF group were randomly submitted to the downhill running and dietary regulation protocol for 8 weeks, which were randomly assigned to four groups: obesity-feeding HFD-sedentary (OHF-Sed group, *n* = 14), obesity-feeding HFD-exercise (OHF-Ex group, *n* = 16), obesity-feeding SCD-sedentary (OSC-Sed group, *n* = 14), and obesity-feeding SCD-exercise (OSC-Ex group, *n* = 16). Serum samples and epididymal fat were obtained at 12 and 20 weeks.

Figure 1 shows the overall design of this study. Male C57BL/6H mice at 5–6 weeks, free-fed individually in a room maintained at 22–25°C on a 12 h day-night cycle with free access to food and water, were purchased from the Charles River Laboratory (Beijing, China). After 1 week of acclimatization, animals were randomly fed one of two diets for 12 weeks: SCD (SC group, *n* = 12, 12% of energy from fat) or HFD (HF group, *n* = 72, 60% of energy from fat). The diets were purchased from the Huafukang Co., Ltd. (Beijing, China), and the mineral and vitamin contents of the two diets were identical. Data of animal food consumption have been previously published (Luo et al., 2020). Twelve mice in each group were randomly sacrificed to detect the effects of the HFD. The number of animals assigned to each diet group was calculated with power analyses using means of adipose IL-10 contents in our preliminary study: 0.5 as significance criterion (two-tailed), 6.2 as expected difference, 2.6 as estimated standardization, and 0.8 as desired power.

The remaining mice in the HF group (*n* = 60) were subsequently assigned to one of four treatment protocols for 8 weeks: obesity-feeding HFD-sedentary (OHF-Sed group, *n* = 14), obesity-feeding HFD-exercise (OHF-Ex group, *n* = 16), obesity-feeding SCD-sedentary (OSC-Sed group, *n* = 14), and obesity-feeding SCD-exercise (OSC-Ex group, *n* = 16). The number of animals assigned to each group was calculated with power analyses using means of adipose IL-10 contents in our preliminary study: 0.5 as significance criterion (two-tailed), 5.5 as minimum expected difference, 2.9 as estimated standardization, and 0.8 as desired power.

All experimental procedures were performed in accordance with the Beijing Sport University Institutional Animal Care and Use Committee-approved protocols and the animal care standards of the American College of Sports Medicine (Animal ethical approval reference number: 2019025A).

### Exercise Protocol

All obese mice in the exercise group were subjected to 3-day acclimation training before beginning the exercise. The protocol for acclimation training is 10 m/min for 15 min with an incline of 0° on the first day, 15 m/min for 15 min with an incline of 0° on the second day, and 15 m/min for 15 min with an incline of -5° on the third day. After the 3-day acclimatization period, obese mice in the exercise group ran on a motor treadmill (-5° slope) at 18 m/min (45% of peak running speed), 1 h/day, 6 days/week for 8 weeks (Granatiero et al., 2017; Dehghani et al., 2018; Chen Y. et al., 2019). The same protocol was used for 8 weeks to avoid any adverse effects, such as skeletal muscle injury. Given that ground reaction impact forces associated with running down such grades may induce uncontrolled inflammation, we intentionally avoided steeper downhill grades (Breiner et al., 2019). Training interventions were conducted between 6:00 p.m. and 9:00 p.m. At the same time (between 6:00 p.m. and 9:00 p.m.), sedentary animals were placed on the other stationary treadmill for 1 h/day, 6 days/week, for 8 weeks to provide a similar environment. As mice generally responded to a gentle tap on the tail or hindquarters, cotton sticks were used to encourage treadmill running.

### Sample Collection

At 12 weeks, animals from the SC and HF groups (*n* = 12) were fasted for 12 h before they were deeply anesthetized (sodium pentobarbital, i.p., 150 mg/kg), and blood samples were obtained from mice orbits for biochemical analyses. Epididymal fat was removed from both sides of the animal and weighed. Epididymal fat pads can be used to represent visceral adipose tissue in the evaluation of obesity, which is more harmful than subcutaneous fat in inducing chronic metabolic diseases and is also more sensitive in response to exercise (Yao et al., 2017).

At 20 weeks, after an 8-week period of downhill running and dietary regulation, animals in the fasting state (12 h) were deeply anesthetized 48 h after the last exercise session, samples were collected as described above.

Half of the mice in each group received an i.p. injection of insulin (0.75 U/kg) 15 min before sample collection, and the other mice were given a PBS injection as control.

### Hematoxylin and Eosin (H&E) Staining for Histomorphological Analysis

**FIGURE 2:**
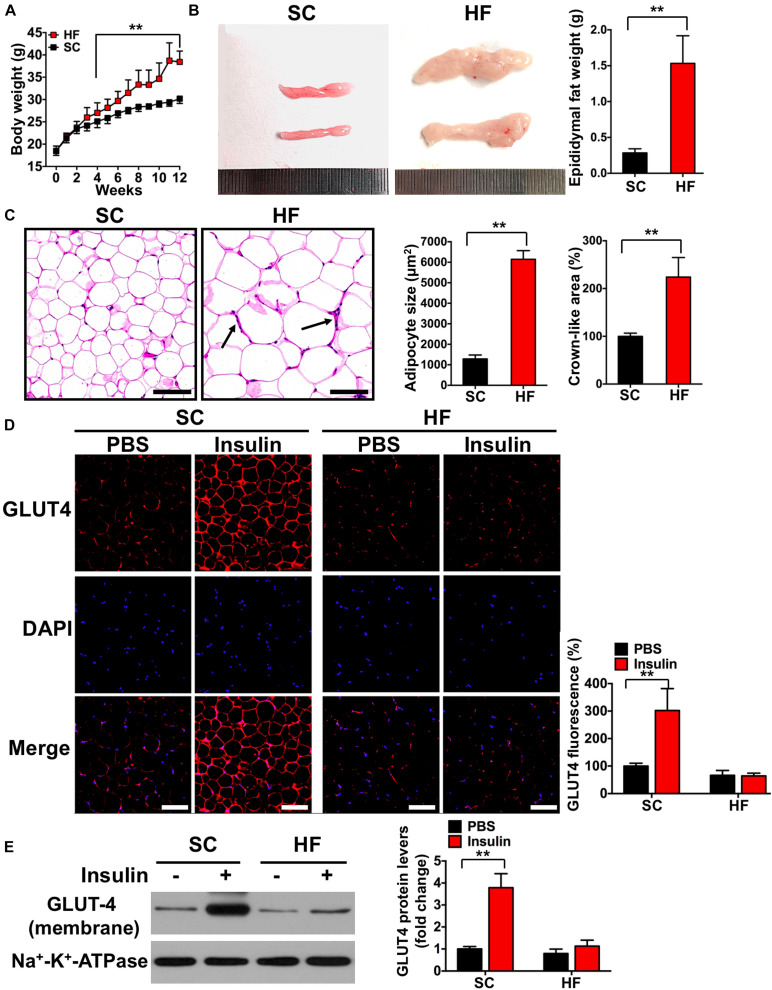
High-fat diet induces insulin resistance in adipose tissue. **(A)** Body weights during 12-week feed. **(B)** Representative image and its weight of epididymal adipose tissue. **(C)** Hematoxylin eosin (H&E) staining of adipose tissue and the areas of adipocyte (scale bar = 100 μm). Black arrows indicate crown-like areas of inflammatory cell infiltration, which are typical manifestations of obesity-induced inflammation. **(D)** Both basal and insulin-stimulated GLUT4 distribution in adipose tissue detected by immunofluorescence (scale bar = 100 μm). **(E)** Both basal and insulin-stimulated GLUT4 protein contents in the adipocyte membrane detected by Western blot. SC, standard control diet; HF, high-fat diet. Values are means ± SD (*n* = 6 per group). **p < 0.01 vs. SC group **(A–C)** or PBS group **(D,E)**. *P*-values were calculated by repeated-measures ANOVA **(A)** or two-tailed Student’s *t*-test **(B–E)**.

The harvested adipose tissues were mounted on a specimen holder containing OCT compound (SAKURA, United States) and frozen in isopentane. Then, 7-μm thick frozen adipose sections (Leica CM 1850, Germany) were stained with H&E staining. Tissue from six mice in each group were used, and more than eight fields were chosen and analyzed for each adipose section. The adipocyte size and crown-like area were determined with Image Pro Plus 6.0 (Media Cybernetics, United States). The differences in crown-like area are displayed as a relative percent normalized to the respective control group (SC group in Figure 2 and OHF-Sed group in Figure 4).

### Immunofluorescence

**FIGURE 3:**
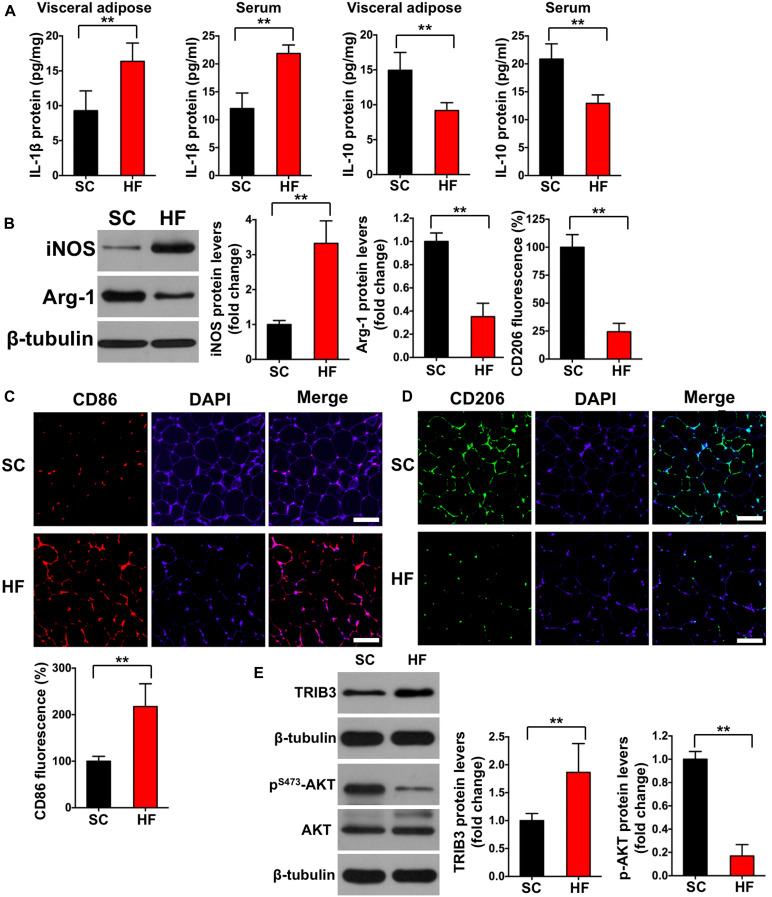
High-fat diet induces chronic inflammation, M1 macrophage activation, and increased TRIB3-AKT signaling. **(A)** IL-1β and IL-10 levels in adipose tissue and serum by ELISA. **(B)** iNOS and Arg-1 protein contents in adipose tissue detected by Western blot. **(C)** CD86 fluorescence in adipose tissue detected by immunofluorescence (scale bar = 100 μm). **(D)** CD206 fluorescence in adipose tissue detected by immunofluorescence (scale bar = 100 μm). **(E)** The protein contents of TRIB3, p-AKT, and AKT in adipose tissue detected by Western blot. SC, standard control diet; HF, high-fat diet. Values are means ± SD (*n* = 6 per group). **p < 0.01 vs. SC group. *P*-values were calculated by two-tailed Student’s *t*-test.

**FIGURE 4:**
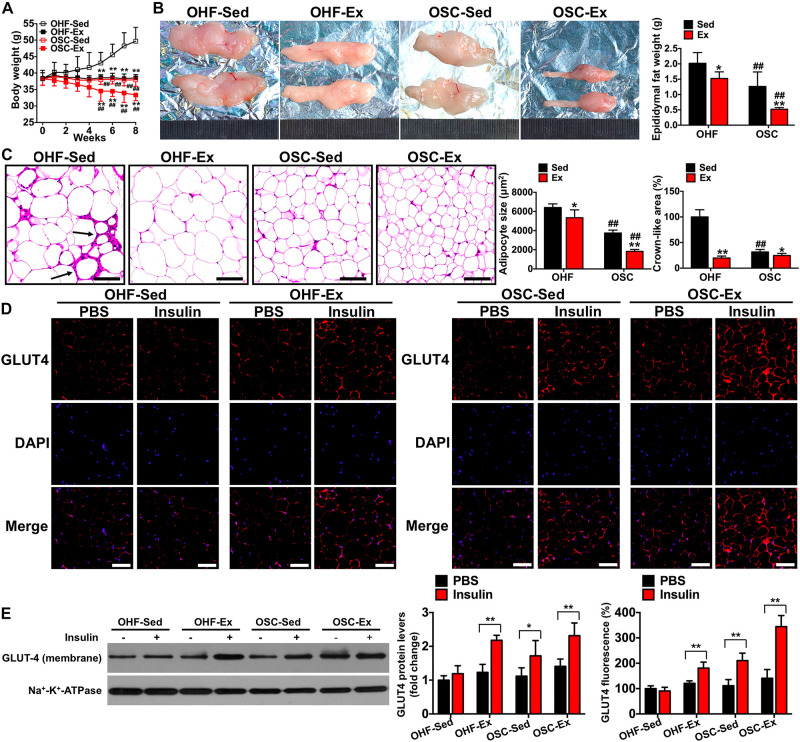
Downhill running and dietary regulation reduces body weight, adipose tissue weight, cross-sectional area, and inflammatory cell infiltration, and attenuates insulin resistance. **(A)** Body weights during the 8-week treatment of downhill running and dietary restriction. **(B)** Representative image and weight of epididymal adipose tissue. **(C)** H&E staining of adipose tissue and the cross-sectional areas of adipocytes (scale bar = 100 μm). Black arrows indicate crown-like areas of inflammatory cell infiltration, which are typical manifestations of obesity-induced inflammation. **(D)** Both basal and insulin-stimulated GLUT4 distribution in adipose tissue detected by immunofluorescence (scale bar = 100 μm). **(E)** Both basal and insulin-stimulated GLUT4 protein contents in the adipocyte membrane detected by Western blot. OHF-Sed, obesity—high-fat diet—sedentary; OHF-Ex, obesity—high-fat diet—exercise; OSC-Sed, obesity—standard control diet—sedentary; OSC-Ex, obesity—standard control diet—exercise. Values are means ± SD (*n* = 6–8 per group). **(A–C)** *p < 0.05, **p < 0.01 vs. corresponding sedentary group; ^##^*p* < 0.01 vs. corresponding OHF group. **(D,E)** *p < 0.05, **p < 0.01 vs. PBS group. *P*-values were calculated by repeated-measures ANOVA **(A)**, two-way ANOVA **(B,C)**, or two-tailed Student’s *t*-test **(D,E)**.

**FIGURE 5:**
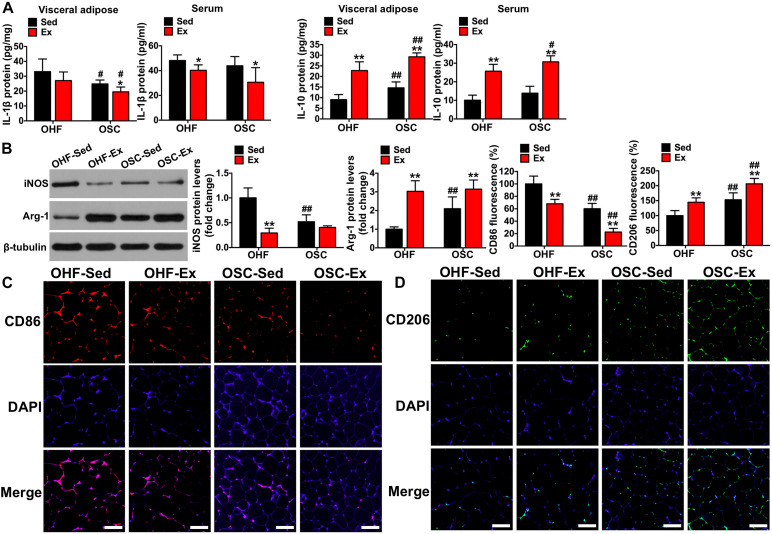
Downhill running and dietary regulation reduces inflammation and activates macrophage M2 polarization in adipose tissue. **(A)** IL-1β and IL-10 levels in adipose tissue and serum by ELISA. **(B)** The protein contents of iNOS and Arg-1 in adipose tissue detected by Western blot. **(C)** CD86 fluorescence in adipose tissue detected by immunofluorescence (scale bar = 100 μm). **(D)** CD206 fluorescence in adipose tissue detected by immunofluorescence (scale bar = 100 μm). OHF-Sed, obesity—high-fat diet—sedentary; OHF-Ex, obesity—high-fat diet—exercise; OSC-Sed, obesity—standard control diet—sedentary; OSC-Ex, obesity—standard control diet—exercise. Values are means ± SD (*n* = 6–8 per group). *p < 0.05, **p < 0.01 vs. corresponding sedentary group; ^#^*p* < 0.05, ^##^*p* < 0.01 vs. corresponding OHF group. *P*-values were calculated by two-way ANOVA.

Frozen adipose sections were prepared as above and incubated with the primary antibody mixture of GLUT4 (ab654, Abcam, United States), CD86 (ab119857, Abcam, United States), or CD206 (ab8918, Abcam, United States) at 4°C overnight. Then slides were protected from light and incubated with secondary Alexa Fluor 488- or Alexa Fluor 555-labeled goat anti-rabbit or mouse antibodies (A-11034, A-21422, Molecular Probes, United States) for 2 h at room temperature. Counterstaining was followed with DAPI for 15 min in an aluminum foil-covered box. Finally, the stained tissues were examined under a fluorescence microscope (Leica, Germany). Six mice for each group were used, and more than eight fields were chosen and analyzed for each adipose section. The fluorescence index was determined with Image Pro Plus 6.0 (Media Cybernetics, United States). The differences in fluorescence are displayed as a relative percent normalized to the respective control group (SC-PBS group in Figure 2, SC group in Figure 3, OHF-Sed-PBS group in Figure 4, and OHF-Sed group in Figure 5).

### Western Blot Analysis for Adipose Tissue

**FIGURE 6:**
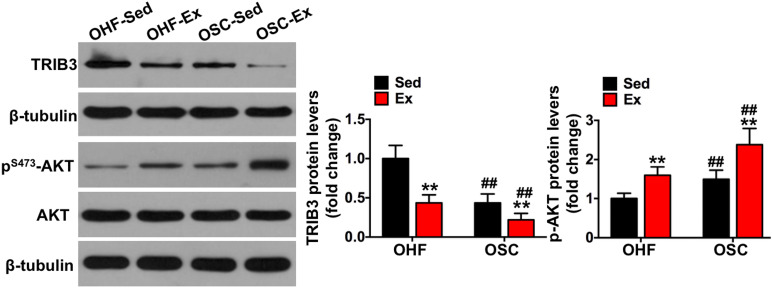
Downhill running and dietary regulation reduces TRIB3-AKT signaling in adipose tissue. The protein contents of TRIB3, p-AKT, and AKT in adipose tissue by Western blot. OHF-Sed, obesity—high-fat diet—sedentary; OHF-Ex, obesity—high-fat diet—exercise; OSC-Sed, obesity—standard control diet—sedentary, OSC-Ex, obesity—standard control diet—exercise. Values are means ± SD (*n* = 6–8 per group). **p < 0.01 vs. corresponding sedentary group; ^##^*p* < 0.01 vs. corresponding OHF group. *P*-values were calculated by two-way ANOVA.

Total protein was extracted using pre-cooled RIPA buffer (Beyotime, China). Membrane proteins to detect GLUT4 were extracted using a Mem-PER Eukaryotic Membrane Protein Extraction Reagent Kit (Thermo Scientific, 89826, United States) according to the manufacturer’s instructions. Protein concentrations were detected using the bicinchoninic acid (BCA) protein assay kit (Thermo Scientific, United States). The samples were then boiled with loading buffer at 98°C for 10 min. A total of 30 μg of protein was electrophoresed on 8–12% SDS-polyacrylamide gel and transferred to a polyvinylidene difluoride membrane (Cell Signaling, United States). The membrane was blocked in 5% bovine serum albumin for 2 h and then incubated at 4°C overnight with primary antibodies, including GLUT4 (ab654, Abcam, United States), Na^+^-K^+^-ATPase (ab76020, Abcam, United States), iNOS (ab15323, Abcam, United States), Arg-1 (ab60176, Abcam, United States), AKT (9272, CST, United States), p-AKT (4060, CST, United States), TRIB3 (ST1032, Millipore, United States), and β-tubulin (YM3030, Immunoway, CN). The membranes were washed with TBST and incubated with HRP-conjugated goat anti-mouse or anti-rabbit secondary antibodies for 2 h the next day (7076, 7074, CST, United States). The membranes were again washed and then exposed using a ChemiDoc XRS imaging system (Bio-Rad, United States). Immobilon Western Chemiluminescent HRP substrate (Millipore, United States) was used to produce a signal to visualize the protein bands. The protein levels within whole-cell and cell membrane lysates were normalized against the levels of β-tubulin and Na^+^-K^+^-ATPase, respectively, and p-AKT protein content was related to total AKT. The captured images were analyzed using Image-Pro plus 6.0 (Media Cybernetics, Inc., United States). The fold changes in protein content are displayed as a relative value normalized to the respective control group (SC-PBS group in Figure 2, SC group in Figure 3, OHF-Sed-PBS group in Figure 4, and OHF-Sed group in Figures 5, 6).

### Enzyme-Linked Immunosorbent Assay (ELISA) for Adipose Tissue and Serum

After visceral adipose samples were collected, total protein was extracted using pre-cooled RIPA buffer (Beyotime, China) at 4°C to detect the levels of IL-1β (MLB00C, R&D, United States) and IL-10 (ab108870, Abcam, United States) by an ELISA kit. After 30 min standing of mice blood, serum samples were obtained by 3,500 *g* centrifugation for 15 min at 4°C. The levels of IL-1β (MLB00C, R&D, United States) and IL-10 (ab108870, Abcam, United States) were measured by the ELISA kit.

### Statistical Analysis

Data are reported as means ± SD. The Shapiro-Wilk test was used to test for normality within each dataset. Non-normal data or unequal variance were log transformed. The repeated-measures ANOVA was used to analyze the differences of body weight. The two-tailed Student’s *t*-test was used for comparison between two groups, and multi-group comparisons were performed with the two-way ANOVA test to analyze the interaction between exercise and diet followed by a Bonferroni *post hoc* test. Statistical analyses were performed using SPSS statistical software, version 20.0 (IBM, Inc.). For all analyses, a *P*-value of 0.05 was considered to be statistically significant. In this study, the interactions between exercise and diet for each index were significant (*p* < 0.05).

## Results

### HFD Induces IR in Adipose Tissue by TRIB3-AKT-Activated Macrophages M1 Polarization

Mice fed a 60% HFD had a significantly higher body weight, epididymal adipose weight, and adipocyte cross-sectional areas when compared with the SC group at 12 weeks (*p* < 0.01; Figures 2A–C). To test the effect of HFD on insulin sensitivity in adipose tissue, we detected both basal and insulin-stimulated GLUT4 fluorescence using immunofluorescence staining. As shown in Figure 2D, insulin-stimulated GLUT4 fluorescence was significantly increased in the SC group (*p* < 0.01), whereas insulin failed to increase GLUT4 fluorescence in the HF group (*p* > 0.05). Consistent with whole-cell GLUT4 fluorescence, insulin stimulation led to a significant increase of GLUT4 protein content on adipocyte membranes (*p* < 0.01; Figure 2E), whereas insulin also failed to increase GLUT4 protein content on adipocyte membranes in the HF group (*p* > 0.05). These data demonstrate that HFD treatment for 12 weeks induces IR in the adipose tissue of mice. The crown-like areas of inflammatory cell infiltration were observed in the adipose tissue of the HF group (*p* < 0.01, Figure 2C, indicated with black arrows), which are typical manifestations of obesity-induced inflammation. To investigate how HFD-induced adipose tissue IR altered systemic and local inflammation, certain cytokines and M1 or M2 macrophage markers were measured. As shown in Figure 3A, mice fed with a 60% HFD had significantly increased IL-1β levels and decreased IL-10 levels both in adipose tissue and serum (*p* < 0.01). Similarly, iNOS protein content and CD86 fluorescence were significantly higher in HF mice compared with SC mice (*p* < 0.01; Figures 3B,C), and Arg-1 protein content and CD206 fluorescence were significantly lower in HF mice (*p* < 0.01; Figures 3B,D). To research potential molecular mechanisms involved in the activation of M1 macrophages in response to a HFD, adipose tissue protein levels of TRIB3, AKT, and p-AKT were assessed. TRIB3 protein content was significantly increased in the HF group compared to the control group, and p-AKT protein content was significantly decreased (*p* < 0.01; Figure 3E), indicating that a HFD for 12 weeks is able to activate TRIB3-AKT signaling in adipose tissue.

### Downhill Running and Dietary Fat Regulation Attenuates IR in Adipose Tissue

To test the influence of combined downhill running training and dietary fat regulation on obesity and insulin sensitivity, we examined body weight, adipose tissue weight, and change in GLUT4 protein content following the 8-week treatment period. As presented in Figure 4, mice treated with downhill running or dietary fat regulation alone (OHF-Ex and OSC-Sed groups) had significantly lower body weights, epididymal fat weights, and adipocyte cross-sectional areas when compared to OHF-Sed mice (*p* < 0.05; Figures 4A–C). The combination of downhill running and dietary fat regulation (OSC-Ex group) resulted in greater decreases in body weight, epididymal fat weight, and adipocyte cross-sectional areas when compared with the OHF-Ex group (*p* < 0.01) and the OSC-Sed group (*p* < 0.01). Insulin failed to increase GLUT4 fluorescence and protein content on the adipocyte membranes of OHF-Sed mice (*p* > 0.05; Figures 4D,E), whereas, the action of insulin to stimulate GLUT4 content on the adipocyte membrane was restored when treated with downhill running and/or dietary fat regulation (OHF-Ex, OSC-Sed, and OSC-Ex mice; *p* < 0.01).

### Downhill Running and Dietary Fat Regulation Reduces Inflammation Level in Visceral Adipose and Serum

As shown in Figure 4C, the crown-like areas of inflammatory cell infiltration were less observed in the adipose tissue of mice treated with downhill running and dietary fat regulation (*p* < 0.05). To further explore the influence of the 8-week treatment of downhill running and dietary fat regulation on inflammation, we detected the levels of IL-1β and IL-10 both in adipose tissue and serum. As presented in Figure 5A, mice treated with downhill running (OHF-Ex group and OSC-Ex group) had significantly higher IL-10 protein contents in adipose tissue and serum, and lower IL-1β in serum, when compared with their respective control groups (*p* < 0.05); mice treated with dietary fat regulation (OSC-Sed group and OSC-Ex group) had significantly lower IL-1β protein contents and higher IL-10 protein contents in adipose tissue when compared with their respective control groups (*p* < 0.05). These data demonstrate that treatments of downhill running and dietary fat regulation for 8 weeks reduced inflammation in HFD-induced IR mice.

### Downhill Running and Dietary Fat Regulation Activates M2 Macrophages in Adipose Tissue

To illustrate the involvement of the macrophage phenotype in the influence of downhill running and dietary fat regulation on HFD-related inflammation, we determined the related markers of macrophage polarization. Consistent with the changes of inflammation, mice assigned to downhill running training or dietary fat regulation alone (OHF-Ex group and OSC-Sed group) had significant reductions in iNOS protein content and CD86 fluorescence, and significant increases in Arg-1 protein content and CD206 fluorescence, when compared to OHF-Sed mice (*p* < 0.01; Figures 5B–D). The combination of downhill running and dietary fat regulation (OSC-Ex group) further improved the macrophage phenotype (inhibited M1 phenotype and activated M2 phenotype) compared with the OHF-Ex group (*p* < 0.01) and the OSC-Sed group (*p* < 0.01).

### Downhill Running and Dietary Fat Regulation Reduces TRIB3-AKT Signaling in Adipose Tissue

To further explore the involvement of TRIB3-AKT signaling on the influence of downhill running and dietary fat regulation on HFD-related macrophage polarization and inflammation, we determined the protein contents of TRIB3-AKT signaling. As shown in Figure 6, TRIB3 protein content was significantly decreased in the adipose tissue of mice assigned to either downhill running training or dietary fat regulation (OHF-Ex group and OSC-Sed group) when compared to OHF-Sed mice (*p* < 0.01), and p-AKT protein content was significantly increased (*p* < 0.01). The combination of downhill running and dietary fat regulation (OSC-Ex group) showed a greater reduction of TRIB3 protein content and a greater increase of p-AKT protein content compared with the OHF-Ex group (*p* < 0.01) and the OSC-Sed group (*p* < 0.01). These results indicate that TRIB3-AKT signaling may be relevant to the regulation of the adipose tissue macrophage phenotype.

## Discussion

Macrophage infiltration into adipose tissue and the resulting inflammation are known to be significant contributors to IR associated with obesity (Castoldi et al., 2016; Gómez-Hernández et al., 2016; Appari et al., 2018). Exercise training accompanied by dietary regulation provides effective therapeutic treatment to attenuate adipose tissue IR induced by HFD-associated obesity. However, the clear mechanism by which exercise training improves insulin sensitivity in adipose tissue remains unclear (Gómez-Hernández et al., 2016; Kubota et al., 2018). The current investigation used downhill running accompanied by a low-fat diet to explore whether the potential mechanism of exercise training and dietary regulation may attenuate adipose tissue IR and inflammation. Novel results from this study indicate that the recovery of insulin signaling in adipose tissue after exercise training and dietary regulation in obese mice may be associated with a decrease in TRIB3-AKT signaling and an increase in M2 macrophages. These findings may help to design better non-pharmacological intervention programs for IR individuals, and be beneficial for searching for potential targets for IR treatment.

In this study, 12 weeks of a HFD attenuated insulin-stimulated GLUT4 translocation and elicited IR in adipose tissue. This was associated with increased adipocyte size, macrophage infiltration and M1 polarization (increased CD86 positive cells and iNOS protein content, and decreased CD206 positive cells and Arg-1 protein content), and inflammation (increased IL-1β level and decreased IL-10 level in adipose tissue and serum). These data confirm results of most studies (Amano et al., 2014; Pincu et al., 2015; Appari et al., 2018) that adipocyte hypertrophy induces macrophage infiltration, M1 polarization, and subsequent chronic inflammation, which elicit IR.

It has been well accepted that exercise training accompanied by a low-fat diet is an effective method for the treatment of IR induced by HFD-associated obesity. In this study, insulin-stimulated GLUT4 translocation was recovered after downhill running and/or dietary regulation for 8 weeks. Simultaneously, we observed that the adipose tissue of mice with HFD-induced adipose tissue IR displayed crown-like areas of inflammatory cell infiltration, which are typical manifestations of obesity-induced inflammation (Murano et al., 2013). However, observational analysis suggested that these crown-like areas were reduced after 8 weeks of combined downhill running and dietary fat regulation. These results suggest that the combination of downhill running and dietary fat regulation may improve adipose tissue insulin sensitivity through a reduction in local inflammation.

Yang and colleagues (Yang et al., 2019) reported that exercise ameliorates adipose tissue inflammation in HFD-induced obese mice, which may contribute to the improvement in the FGF21-adiponectin axis impairment. Peppler et al. (2017) found that habitual physical activity can attenuate the LPS-induced inflammatory response in adipose tissue by reducing mRNA expression of MCP-1 and IL-6. Kawanishi et al. (2013) suggested that exercise training reduces adipose tissue inflammation by suppressing infiltration of inflammatory macrophages and CD8 T cells. The results from this study suggested that downhill running and dietary regulation reduces inflammation by decreasing pro-inflammatory IL-1β and elevating anti-inflammatory IL-10 in both adipose tissue and serum. Although acute eccentric exercise is usually used to induce inflammation in skeletal muscle, the inflammatory response induced by exercise usually undergoes a switch process from pro-inflammation to anti-inflammation. Zuo et al. (2018) detected the early invasion of pro-inflammatory M1 macrophages at 6 h after eccentric exercise. However, anti-inflammatory M2 macrophages were detected in muscles before 24 h after exercise, which even denied the entire pro-inflammatory M1 phenotype during the early stages of tissue repair. We collected samples 48 h after the last exercise. In addition, exercise-induced inflammation is commonly induced by high-force, unaccustomed stretches. In this study, given that the ground reaction impact forces associated with running downhill may induce uncontrolled inflammation, we intentionally avoided steeper downhill grades. During the 8 weeks of chronic training, the mice in the current study adapted to this exercise protocol.

Although it has been well accepted that exercise training is an effective method for treatment of IR induced by HFD-associated obesity, the clear mechanism by which exercise training attenuates adipose tissue inflammation to attenuate IR remains to be elucidated (Macpherson et al., 2015; Emmons et al., 2019; Luo et al., 2020). Given the decreases of inflammation in downhill running and dietary regulation-treated mice, we further interested if exercise training and dietary regulation present similar effects on macrophages (Luo et al., 2020). The significance of the macrophage phenotype to insulin sensitivity was previously demonstrated in a study in which M2-dominant polarization macrophages attenuated HFD-induced inflammation and IR in adipose tissue and the liver (Chen G. et al., 2019). Instead, the M1 macrophage is known to be the central mediator of obesity-induced inflammation and IR, which leads to disruption of glucose homeostasis (Macpherson et al., 2015). Here, the pro-inflammatory M1 macrophage was increased in the adipose tissue of obese mice, which was accompanied by a reduction in the anti-inflammatory M2 macrophage. However, the increase in the M1 macrophage was dampened and M2 macrophage activation was increased after 8 weeks of downhill running and dietary regulation. Accordingly, we conclude that the macrophage phenotype may be related to the influence of downhill running and dietary regulation on adipose tissue IR. Although the results of CD86 and CD206 from immunofluorescence cannot detect the absolute number of macrophages in adipose tissue, as can be done with flow cytometry, we believe that our data provide evidence of relative changes in M1 or M2 macrophages.

TRIB3 is known to be mediator of IR, which disrupts multiple downstream effects of insulin signaling by inhibiting downstream target protein AKT phosphorylation (Zhang et al., 2013; Mondal et al., 2016). Further, Riera-Borrull et al. (2017) reported that TRIB3 mediates palmitate-induced activation of proinflammatory M1 macrophages, which suggests that TRIB3 is relevant to the regulation of the macrophage phenotype. In agreement with this, in this study, TRIB3 was also elevated in the adipose tissue of HFD-induced obese mice, which induced a reduction in AKT phosphorylation, and was further associated with the activation of M1 macrophages and the inhibition of M2 macrophages. Although it has been popularly accepted that TRIB3-AKT signaling has a critical role in regulating IR, there are apparent controversies in the regulation of exercise on TRIB3 signaling. Rodrigues et al. (2015) reported that acute exercise reduced TRIB3 protein levels in the hypothalamus of obese rats. Matos et al. (2010) demonstrated that acute exercise reverses TRIB3 expression and insulin signaling restoration in muscle. Canciglieri et al. (2018), however, reported that TRIB3 content was not altered after short-duration physical exercise with a 10° inclination. In the current study, the increase in TRIB3 was dampened and AKT phosphorylation was increased after 8 weeks of downhill running training and dietary fat regulation. These results suggest that TRIB3-AKT may be related to the influence of downhill running and dietary regulation on the macrophage phenotype in adipose tissue. Namely, exercise in the current study reduced TRIB3 protein level, which directly induced activation of the downstream target protein AKT. Then, activated AKT inhibited macrophages M1 polarization and promoted M2 polarization (Lu et al., 2017), which ultimately improved adipose tissue inflammation and attenuated IR.

In addition, it has been recognized that the dynamic patterns of the macrophage phenotype in skeletal muscle upon eccentric exercise stimuli play a key role in regulating inflammation and promoting skeletal muscle remodeling after eccentric exercise (Zuo et al., 2018). Concurrently, TRIB3 participates in the regulation of macrophage phenotype and function (Wang et al., 2012; Riera-Borrull et al., 2017). Therefore, we hypothesized and demonstrated that downhill running may also regulate adipose tissue macrophage polarization in mice, and TRIB3 signal may participate in this process. A limitation of the present study is that we did not include a group that performed level or uphill running to verify the specific mechanism of eccentric exercise in improving IR, although some researchers have shown that eccentric exercise results in more improvements in glucose tolerance and IR when compared with the concentric intervention (Paschalis et al., 2011; Chen et al., 2017; Julian et al., 2019). In the future, further research is needed to verify how much of the effects of exercise on adipose tissue IR were due to eccentric muscle contractions and what was the specific mechanism. Further, considering the difference of mechanical stress between mice and humans during downhill running, the exercise protocol in this study may be unable to directly apply to humans, and more studies are needed to promote its translation.

In summary, the results from this study reveal that downhill running accompanied by dietary regulation attenuates HFD-related IR in mice, which may be associated with reduced TRIB3-AKT signaling and activation of M2 macrophages in adipose tissue. Specifically, our results demonstrate that HFD upregulates TRIB3-AKT signaling, activates macrophages M1 polarization, and induces IR in adipose tissue. Whereas, downhill running and dietary regulation attenuates HFD-related IR, reduces TRIB3-AKT signaling, and activates macrophage M2 polarization in adipose tissue. This study adds new and important evidence for further searching for potential targets for IR treatment and may help to design better non-pharmacological intervention programs for IR individuals.

## Data Availability Statement

The raw data supporting the conclusions of this article will be made available by the authors, without undue reservation.

## Ethics Statement

The animal study was reviewed and approved by the Animal Care and Use Committees of Beijing Sport University.

## Author Contributions

WL, YZ, and QT conceived the study and designed the experiments. WL and LA wrote the manuscript. WL, LA, and YZ carried out the experiments and contributed to sample collection. LA analyzed the data. All authors contributed to the study and have read and approved the final version.

## Conflict of Interest

The authors declare that the research was conducted in the absence of any commercial or financial relationships that could be construed as a potential conflict of interest.
